# Evaluation of New Technology-Based Tools for Dietary Intake Assessment—An ILSI Europe Dietary Intake and Exposure Task Force Evaluation

**DOI:** 10.3390/nu11010055

**Published:** 2018-12-28

**Authors:** Alison L. Eldridge, Carmen Piernas, Anne-Kathrin Illner, Michael J. Gibney, Mirjana A. Gurinović, Jeanne H.M. de Vries, Janet E. Cade

**Affiliations:** 1 Nestlé Research, Vers-chez-les-Blanc, 1000 Lausanne 26, Switzerland; 2 Nuffield Department of Primary Care Health Sciences, University of Oxford, Oxford OX2 6GG, UK; carmen.piernas-sanchez@phc.ox.ac.uk; 3 College of Health Sciences, Polytechnic Institute UniLaSalle Beauvais, 60026 Beauvais, France; Anne-Kathrin.ILLNER@unilasalle.fr; 4 Institute of Food and Health, University College Dublin, Dublin D04 V1W8, Ireland; mike.gibney@ucd.ie; 5 Centre of Research Excellence in Nutrition and Metabolism, Institute for Medical Research, University of Belgrade, Belgrade 11000, Serbia; mirjana.gurinovic@gmail.com; 6 Division of Human Nutrition and Health, Wageningen University, 6708WE Wageningen, The Netherlands; jeanne.devries@wur.nl; 7 School of Food Science and Nutrition, University of Leeds, Leeds LS2 9JT, UK; J.E.Cade@leeds.ac.uk

**Keywords:** dietary assessment, mobile technologies, Web-based technologies

## Abstract

Background: New technology-based dietary assessment tools, including Web-based programs, mobile applications, and wearable devices, may improve accuracy and reduce costs of dietary data collection and processing. The International Life Sciences Institute (ILSI) Europe Dietary Intake and Exposure Task Force launched this project to evaluate new tools in order to recommend general quality standards for future applications. Methods: A comprehensive literature search identified technology-based dietary assessment tools, including those published in English from 01/2011 to 09/2017, and providing details on tool features, functions and uses. Each of the 43 tools identified (33 for research and 10 designed for consumer use) was rated on 25 attributes. Results: Most of the tools identified (79%) relied on self-reported dietary intakes. Most (91%) used text entry and 33% used digital images to help identify foods. Only 65% had integrated databases for estimating energy or nutrients. Fewer than 50% contained any features of customization and about half generated automatic reports. Most tools reported on usability or reported validity compared with another assessment method (77%). A set of Best Practice Guidelines was developed for reporting dietary assessment tools using new technology. Conclusions: Dietary assessment methods that utilize technology offer many advantages for research and are often preferable to consumers over more traditional methods. In order to meet general quality standards, new technology tools require detailed publications describing tool development, food identification and quantification, customization, outputs, food composition tables used, and usability/validity testing.

## 1. Introduction

The opportunities provided by the internet to link large scale food and nutrient databases with automated dietary recording has led to growth in the number of online dietary assessment tools [1]. New technologies for measuring diet can be categorized according to the type of technology being used, such as Web-based or online tools, mobile systems (apps), camera-based tools, and other developing technologies, such as consumer purchase data and wearable sensors. Traditional methods relied heavily on self-reporting of foods consumed either using food frequency questionnaires (FFQ) or with paper-based recalls or diaries. All of the traditional methods lacked accuracy as a result of problems including the ability to recall food consumed, difficulties with portion size estimations or limited food composition tables [2]. Considerable manual input and time was required for coding and converting foods recorded into nutrients. This meant that in large-scale cohort studies it was not generally possible to collect detailed food intake information, and studies relied on food frequency questionnaire data, which is subject to greater measurement error than other self-report measures [3,4]. Use of computerized tools facilitated data coding, and incorporation of the automated multiple-pass method (AMPM) standardized data collection for national surveys [5,6]. New methods have allowed for an expansion and potential improvement on the traditional methods. The use of the Internet makes larger-scale collection of food and nutrient information practical with lower costs and burden for both researchers and participants [7]. Study participants can be invited to take part in research electronically via email or text [8]. Users of new technology tools can more easily identify foods consumed through interactive searchable databases [9]. They can provide real-time results and feedback [1] and can include enhanced options for portion size description, such as using digital images [10], and more relevant lists of branded food items [9].

It is often not clear how relevant a particular dietary assessment tool is for research as a result of limited information provided on the development process and lack of validation. An evaluation of new technologies to assess diet may help understanding of their potential to replace, improve, or complement traditional methods. Due to the rapid development of new technologies, existing reviews of the area quickly become out of date, including obsolete technologies such as personal digital assistants or PDAs [11]. Highlighting features of new technologies, such as those found in Web-based recalls or apps, in comparison with tool elements reflecting traditional approaches may help to identify techniques that can enhance dietary measurement [12]. Recently, clear guidance in terms of dietary assessment tool choice and reporting has been published [2,13]. However, guidance on the development of new tools with quality criteria for their assessment is still lacking.

In 2016, the International Life Sciences Institute (ILSI) Europe Dietary Intake and Exposure Task Force (http://ilsi.eu/task-forces/food-safety/dietary-intake-and-exposure/) established an expert group on evaluation of new methods for dietary intake assessment. The aim of the group was to review new technologies for diet assessment in terms of features, sources and quality of data, and validity. The review presented here will help to understand the relative merits of particular new tools and applications currently available for dietary intake assessment. We have critically evaluated tools, including their sources of data, applicability for research, ease of use by different population groups, and ability to handle a wide range of foods and beverages. In a second step, we also suggest guidelines for quality standards to improve reporting of dietary intake assessment tools.

The objectives of this paper are to: (i) report on a comprehensive review of tools for dietary assessment using new technologies which are applicable for use in research, commercial, clinical and public health contexts; (ii) to develop guidelines for quality criteria required for a good quality tool; and (iii) to make recommendations for future reporting of dietary assessment tools using new technologies.

## 2. Materials and Methods

### 2.1. Inclusion Criteria and Search Strategy

Comprehensive literature searches were conducted to identify articles pertaining to new technologies for dietary intake assessment using key word searches with the following inclusion criteria: (1) publications were in English, (2) articles were published from January 2011 to September 2017, and (3) sufficient information was available to evaluate tool features, functions, and uses. Various search terms were used related to dietary or nutrition surveys, nutrition assessment, and the use of technologies, including mobile apps, Web-based tools, online or Internet tools, and software. PubMed, PLOS, BioMED, Science Direct and Ovid databases were used, each with slightly different search terms (Supplemental Table S1). The searches were limited to articles published after 1 January 2011 because the field of technology development for dietary intake assessment is advancing rapidly, and tools developed prior to 2011 have been previously evaluated [12]. Dietary assessment tools were identified, details of which were available in one or more publications.

### 2.2. Evaluation Criteria and Data Extraction

The Expert Group, comprised of the authors of this manuscript, identified 25 attributes related to data entry, identification and quantification of foods, customization, output, usability and validity, which were used to evaluate each dietary assessment tool (Supplemental Table S2). Under the heading of Data Entry, we assessed whether the tools relied on text entry, digital images and/or bar-code scanners, and whether they also collected information about health characteristics or physical activity. For the Identification and Quantification of Foods, we assessed whether the foods or beverages were automatically identified from an image or required manual identification, the source of food composition data used, and how the intake amounts were quantified, either by weights or household measures, or estimated from digital images. In the Customization section, we assessed whether the tool allowed the user to add missing foods, custom recipes or dietary supplements, and whether the program used machine learning to adapt the list of foods to user preferences. Under Output, we considered whether the tool provided data on energy, macro- and micro-nutrient intakes, food groups consumed, time of intake and meal name, and whether the tool generated automated reports. Finally, we assessed Usability and Validity by checking whether there were any reports of user feedback, time to complete the assessment, and whether any validation studies had been conducted.

The features of each dietary assessment tool were assessed independently by two members of the Expert Group from details provided in the publications, and any discrepancies were discussed at the Expert Group level. If the publications identified in the searches did not provide the sufficient detail to complete the assessment, additional literature, websites, contacts with authors, or tool use itself were used to attempt to fill gaps.

## 3. Results

### 3.1. Search Results

**Figure 1:**
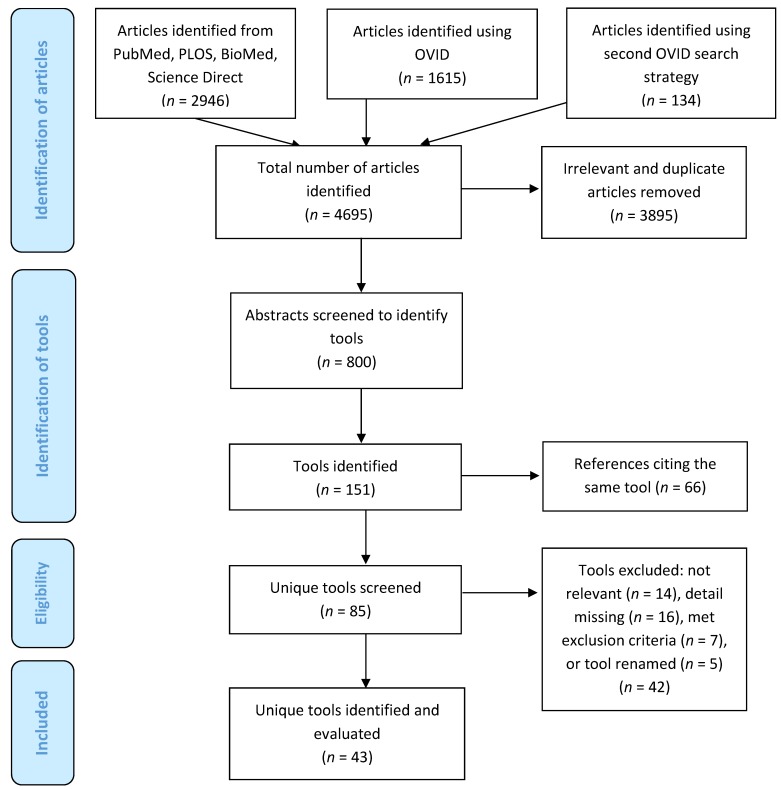
PRISMA diagram used to identify technology-based tools for dietary intake assessment.

The PRISMA diagram showing the search flow and inclusion/exclusion of studies appears in Figure 1. A total of 4695 articles were initially identified. Duplicates were removed and the remaining articles screened (title and abstract) to eliminate those that were not relevant to meet the project objectives, yielding a total of 800 publications related to dietary intake databases, applications, and tools. The goal of this review was to identify unique technology-based tools for dietary intake assessment, including smartphone applications, those that captured digital images of foods and beverages for the purpose of dietary intake assessment, and dietary assessment tools available from the Web or that were accessed from a personal computer (PC). From the 800 articles that mentioned dietary assessment in the title or abstract, 151 were related to new technologies for dietary intake assessment, and of these, 66 were additional references for tools already identified. Papers describing the remaining 85 tools were reviewed in detail. A further 42 were excluded following the detailed review: 14 were deemed to be not relevant because they were editorials (*n* = 1), review papers (*n* = 4), or did not describe a new tool for dietary intake assessment (*n* = 9); 16 were missing sufficient detail to do our evaluation; seven of the tools were developed and reported on prior to 2011, thereby meeting our exclusion criteria; and five were eliminated because the publications referred to a tool that had been subsequently renamed. In the latter case, the updated tool name was retained for our evaluation. Consequently, we included 43 unique tools in our evaluation.

### 3.2. Characteristics of Included Studies

In total, from the 43 tools identified, 33 tools were for use in research or surveillance and 10 tools intended for direct consumer use (Table 1), and since several of the attributes differed between the research/surveillance tools and those designed for consumers, we separated them. Of the 33 tools used for research or surveillance, *n* = 21 (64%) were Web-based to be used on a computer; *n* = 6 (18%) were optimized to be used on smartphones; *n* = 3 (9%) were for PC only (not Web-based); *n* = 2 (6%) used wearables for data collection and *n* = 1 (3%) was designed to be used on a tablet. Of the 10 tools identified for consumer use, *n* = 8 (80%) were optimized for smartphone use and *n* = 2 (20%) were Web-based to be used on a computer. Of the 33 tools designed to collect dietary data for research purposes, *n* = 16 (48%) were designed for adults exclusively, *n* = 11 (33%) were for all ages, and *n* = 6 (18%) were exclusively for children and/or adolescents. Of the 10 tools designed for consumer use, *n* = 7 (70%) were for adults exclusively, while *n* = 3 (30%) were designed for all ages. Among all the tools designed for research purposes, *n* = 17 (52%) collected dietary intake over the previous 24h using dietary recalls; *n* = 11 (33%) collected food records, while the rest collected intakes via food frequency questionnaires (*n* = 3; 9%) or imaging systems (*n* = 2; 6%). Of the 10 tools designed for consumer use, most of them collected food records (*n* = 8; 80%), while *n* = 2 (20%) collected food frequency questionnaires.

Although all of these tools used technology for dietary intake data collection, not all of the tools automatically coded the intake information to generate energy and nutrients (Table 1). Of the tools assessed here, 15 of the 43 (35%) were used for data capture only and required a dietitian or a coder to enter the items and portions in another tool later to estimate energy and nutrient intakes. These are identified as “not integrated into the tool” in Table 1. Another large difference in the tools was the source of food composition data and the number of items available. Tools designed to assess food consumption frequency (Evident II, Food4Me, GraFFS, IDQC, Oxford WebQ, and WebFFQ) included 135–200 individual line items (individual foods or aggregated food categories). Those designed for children varied, with SNAP and WebCaaFE including a limited list (49 and 32 foods and beverages, respectively), while WebFR and WebDASC included a more extensive list of 550 and 1300 items, respectively. Tools that relied on national food composition tables ranged from about 1000 items to more than 45,000 if branded foods were also included (e.g., myfood24), and were largely complete with respect to nutrients. The source of food composition was reported in all but one case, but the number of foods included in the database was missing for six of the tools. The daily time to complete each tool was reported in 18 of the 43 studies. The times ranged from an average low of 14 min to as much as 45–60 min, but most tools were completed within 15–35 min.

The use of images also differed considerably among tools. TADA, Snap-N-Eat, and DietCam automatically coded foods and beverages from digital images [14,15,16], and RFPM used semi-automatic coding of images to facilitate data entry. GoCARB automatically coded carbohydrate content of food categories identified from images. Chest-worn cameras, like eButton or Microsoft SenseCam, captured digital images throughout the day but required subsequent coding by nutritionists for nutrient intake estimates. Several tools, CHAT, FoodNow, NANA, NuDAM, and TECH, used digital images to enhance reporting of food intakes, along with text or voice recordings. FoodLog used images as a visual diary of food intakes for patients with diabetes, and Microsoft SenseCam used images as a memory aid for food records.

### 3.3. Comparison of Tools Used for Research versus Those for Consumer Use

**Figure 2:**
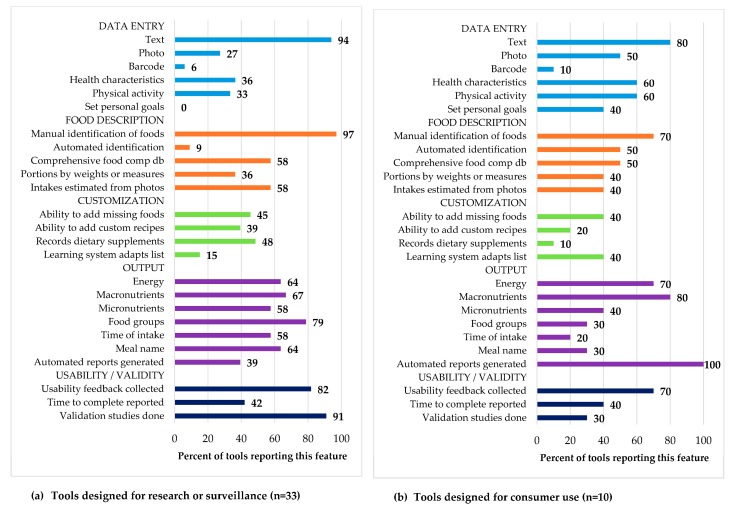
Summary rating of the features from the dietary assessment tools designed for research or surveillance (**A**) and for consumer use (**B**).

Figure 2 compares the 25 attributes evaluated according to use in research (*n* = 33) vs. those intended for consumer use (*n* = 10). The greatest differences in summary ratings occurred in the category ‘Data entry,’ where half of consumer access tools made use of photos for data entry, compared to less than a third of tools used in research or surveillance. In addition, information on health characteristics and physical activity were more prevalent in tools for consumer access (60%, six tools), compared to only 36% (12 tools) and 33% (11 tools) of research or surveillance tools, respectively. The possibility to set personal goals was identified as a unique feature in tools for consumer access. In the category ‘Food description’ differences were observed for the automated identification of foods, in particular, with 50% (5) of consumer access tools offering this functionality, compared to only 9% (3) of research and surveillance tools. With regard to the category ‘Customization,’ research and surveillance tools had proportionally more options to add missing items, customize recipes, and report use of dietary supplements. Research and surveillance tools more frequently provide detailed information on dietary intake in the ‘Output’ category, particularly for the features ‘Food groups’, ‘Time of intake’, and ‘Meal name’, but fewer of the research tools contained integrated food databases, so lacked the ability to estimate energy or nutrient intakes automatically. In contrast, all consumer access tools we identified generated automatic reports, but only 39% (13 tools) of research and surveillance tools did so. In the ‘Usability and validity’ category, a higher proportion in tools used for research or surveillance (91%; 30 tools) have conducted validation studies, compared to 30% (*n* = 3) consumer access tools.

### 3.4. Validation Studies

Some type of validation study was published for 33 of the 43 new technology-based tools evaluated in this review. Seven of the tools compared energy intakes with Total Energy Expenditure (TEE) from doubly-labelled water (DLW) or accelerometers (Supplementary Table S3). In the DLW studies, energy intake estimates from the new technology tools were significantly lower than the TEE in studies using the Microsoft SenseCam [51], NuDAM [58], RFPM [64], and TADA [72] (differences ranging from 750 to 3745 kJ/day (179–895 kcal), whereas a different study with RFPM was within 636 kJ (152 kcal) [64], and two studies in children using the TECH tool were within 220–330 kJ (53–79 kcal) of TEE [74,75]. Two validation studies compared new technologies with TEE estimated from accelerometer data, showing that WebFR underestimated intakes by an average of 1840 kJ (440 kcal) in children 8–14 years [86], and FoodNow underestimated energy by 826 kJ (200 kcal) in young adults [44].

**Figure 3:**
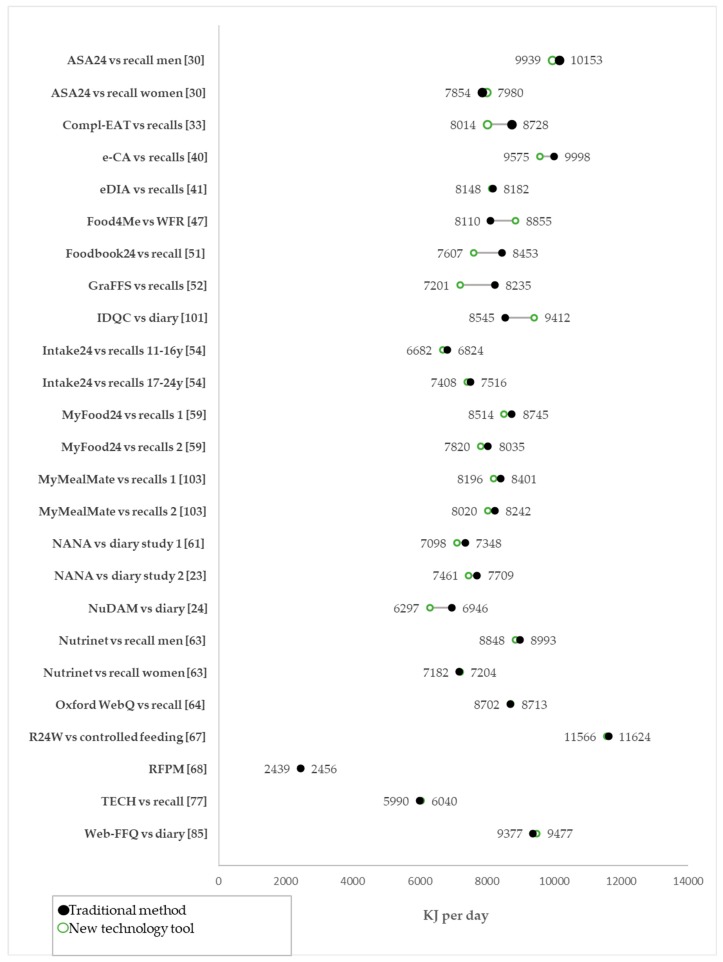
Energy estimations from digital tools vs. traditional methods of dietary intake assessment.

Standard methods of dietary assessment, including 24-h recalls, food records or weighed portions, were used in validation studies for 19 of the new technology tools (representing 25 individual validation comparisons), and in these studies, there was much closer agreement (Figure 3). In fact, 18 of the 25 individual comparisons were within 250 kJ (about 60 kcal) of each other when comparing the tool and the traditional method. Six of the comparisons were within 400–900 kJ (95–215 kcal), and only one had a difference greater than 1000 kJ (240 kcal) compared to the traditional method. The tools NuDAM, RFPM, and TECH were assessed using both DLW and compared with standard method of dietary assessment, e.g., 24-h recall, weighed foods, or a diary.

Macronutrient intake comparisons were available for 22 of the 25 validation comparisons (Supplemental Table S2). Protein intake estimates were the closest between traditional and new technology tools, with 18 comparisons within 5 g of the reference (average 2.1 g). Three of the protein comparisons were between 5–9 g different from the reference and only one was >10 g. Agreement was less accurate for fat with 13 comparisons within 5 g of the reference, four between 5–9 g, and three comparisons >10 g difference. Carbohydrate estimates showed the widest variation, with eight comparisons within 5 g, six between 5–9 g, and eight >10 g.

The remaining 10 tools were validated using some other method. For example, the portions estimated from the eButton were compared to actual volumes measured by seed displacement [31]. WebDASC [80] and Epic-Soft [35] were compared with biomarker data. SNAP [67], SNAPA [68], and WebCAAFE [78] compared reported foods and beverages against observations. Results from a study using DES were compared with results from a national survey in the same population [28], and DAP compared FFQs with 24-h recalls collected using the same tool [26]. VNP was evaluated by comparing the coding of 24-h recalls with DietPro 5i, a different dietary intake coding software [77]. Lastly, GoCARB was compared with self-estimates of carbohydrates and carbohydrate intakes calculated from weighed food samples [100].

## 4. Discussion

The ILSI Dietary Intake and Exposure Task Force initiated this evaluation because of the rapid emergence of technologies available for dietary intake assessment coupled with concerns about a lack of quality standards for their development. Our review was anchored by a previous review and evaluation of innovative technologies for nutritional epidemiology, which assessed publications from 1995–2011 [12]. Since that review was published, personal digital assistants (PDAs) are no longer on the market, tape recorders are no longer needed for voice recording of dietary data, and cameras are integrated into smartphones, making digital image capture of foods much simpler. We focused our review on tools identified from publications in 2011–2017, and only four tools (ASA24, Nutrinet Santé, Oxford WebQ, and RFPM) were included in both this and Illner’s previous assessment.

There is growing pressure in the area of dietary intake assessment to improve the accuracy and reduce costs of data collection and processing [107]. New technology tools use a variety of inputs for dietary assessment, including text, voice, digital images, and bar-code scanners. Various techniques have been implemented to enhance accuracy of portion size reporting, including automatic estimation from digital images and visualization of different sized portions on a plate, as well as the ability to report quantities by weight or common household measures. Many new technology tools, especially those designed for consumer use, provide automated feedback on the individual’s nutrient intakes or dietary patterns, which may improve dietary outcomes and promote behavior change [108,109]. People are now accustomed to using technology tools, like smartphones, tablets, and computers, as part of their daily life, and usability studies indicate that many prefer technology tools for dietary intake assessment over traditional methods [20,42,71,104].

In the meantime, a number of other reviews have been published. While we deliberately chose to focus on new technologies identified from the published academic literature, other reviews have used app-store downloads as the criteria for selection [110,111]. Few of the app-store tools (4%) provided details about the sources of food composition data, and only 14% provided micronutrient estimates [111]. In contrast, half of the consumer apps in our review used a comprehensive food composition table, and 40% reported on micronutrient intakes. It is clear from the two approaches that apps with publications are more likely to include comprehensive food composition databases and, therefore, can report on a full complement of nutrients, compared to the most popular consumer apps.

Image capture can increase accuracy and ease reporting of foods and beverages consumed [14,50]. Images were used for data capture in 13 of the tools we evaluated (nine research and four consumer-based tools), either by automatically coding food intakes, passively capturing food intake throughout the day, as a method of recording intakes, or as a memory prompt. Digital images were also used to facilitate portion size estimation in over half of the tools we evaluated (53%; 19 research tools and four consumer tools). Uses ranged from automatic estimation of food volumes from digital images [14,15,16,30] to visualization of different portion sizes to improve portion-size reporting [20,26,40,42,45,46,52,59,81,83,84,87].

Validation studies were much more commonly reported for dietary assessment tools in the research setting than for those targeted to consumers. There was very good agreement between many of these tools and their reference method, a conclusion also drawn in another previous review [112]. We found that 30 (out of 33) of the research tools and three (out of 10) of the consumer tools conducted a validation study, although the majority of comparison methods used in validation were other self-report measures and, therefore, subject to similar errors. In 72% of the comparisons (18 of 25), the new technology was within 60 kcal of the traditional method of dietary intake assessment. The differences were somewhat wider for studies with DLW, but these differences could have been due to a variety of reasons, including estimate errors from coders manually coding from images, or because eating occasions were not reported. As pointed out previously, new technologies will not resolve all of the challenges of dietary assessment [1], but it is also reassuring that, in many cases, results are close to traditional self-reported or memory-based recalls, which have received recent criticism for their accuracy [113]. Objective biomarkers of dietary intake, such as DLW, urinary nitrogen or potassium, or plasma vitamin levels, are still lacking for most tools [1,112], and care must be taken to interpret validation by other means, such as direct data entry into two comparable tools, or comparison of results from a national survey, for example.

The technology tools we reviewed were developed for use across a wide variety of geographies, including both higher and lower-income countries. Two tools in particular were developed to facilitate interviewer-assisted data collection in lower-middle income countries [54,87], illustrating the utility of technology tools, even in countries where individuals may not have access to a smartphone, personal computer, or other technology for personal monitoring. However, technology tools will have limited use for self-monitoring in countries where smartphone or personal computers are not widely available.

Our evaluation has several notable strengths. As new tools and technologies are constantly changing, we have updated previous reviews with new tools identified from the literature and added a comprehensive evaluation of features. We have also compared features of research-based tools with those designed primarily for consumers, highlighting differences across all of our assessment topics. However, we must also acknowledge limitations in our review. The review was completed in September 2017, and it is possible that more recent publications have not been included in our review. For example, an in-depth validation of myfood24 including biomarkers was published after our assessment was completed [114], and others may have been missed as well. Results from validation studies comparing new technology tools to TEE or with daily energy estimations from conventional methods studies were presented, but further assessment of the quality of those studies was not assessed. We also focused on dietary assessment, per se, and have not included other new methods for assessing intakes, such as bite counters, tools that measure chews and swallows, or wrist-tracking devices that measure feeding [115]. It is also possible that there could be other attributes that are also important, but were not covered in this review, such as ethical issues or privacy when digital devices include other identifying features [111]. The impact of new technologies on cost will depend on the specific study design and the tools used, and this was rarely addressed in any of the publications. Finally, the search strategy may have missed some apps if key word searches did not pick up the studies, however, we used several search engines and different key word searches to minimize this risk.

**Figure 4:**
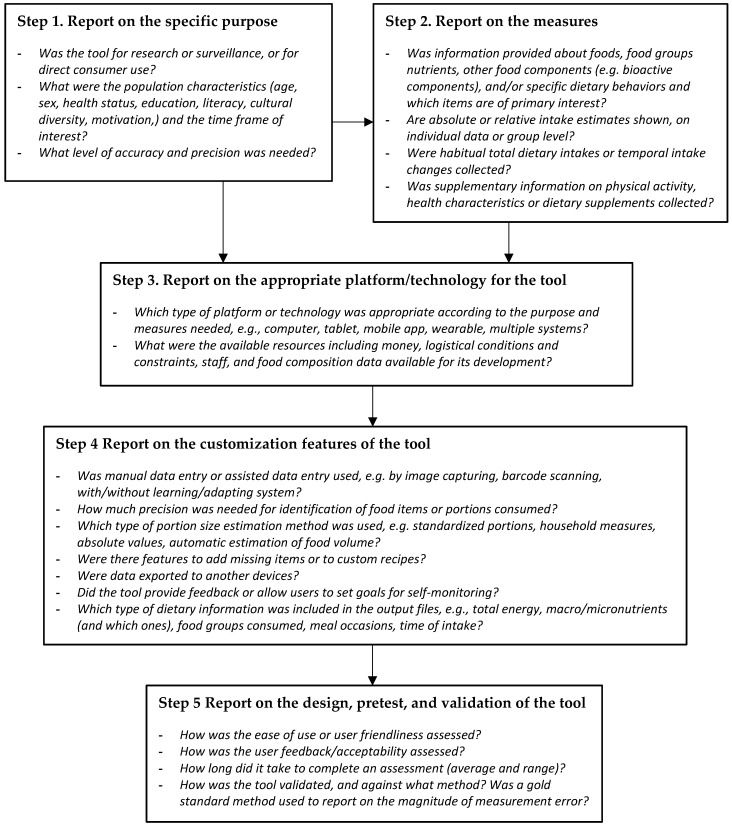
Best practice guidelines for reporting new technologies for dietary assessment.

The quality of tools cannot be assessed if this information is considered to be proprietary, or is omitted from scientific publications. Our assessment included 25 attributes in the areas of data entry, food description, customization, output, and usability/validity. Based on our evaluation of new technology-based tools for dietary intake assessment we have developed best practice guidelines for reporting on new technologies for dietary assessment (Figure 4), which add to existing STROBE-nut guidelines (referring to Strengthening the Reporting of Observational Studies in Epidemiology, for nutrition epidemiology) [13].

### 4.1. Best Practice Guidance for Reporting on New Technologies for Dietary Assessment

#### 4.1.1. Step 1: Report on the Specific Purpose

The goal of the first step is to report on the purpose of the dietary assessment tool. This depends primarily on the context in which the tool has been used. Issues related to the assessment of dietary data needed for research or surveillance purposes may differ from those needed for consumer access settings. Report what you aimed to measure, in what population, and over what period of time. In addition, the definition of the specific purpose of a tool implies the identification of the population characteristics, e.g., age, sex, health status, educational level. It is also important to inform about what level of accuracy and precision was needed. For example, if a higher level of precision was required, it may be necessary to administer repeated measurements.

#### 4.1.2. Step 2: Report on the Measures

The goal of the second step is to inform about the main measurement features of a given tool. These relate to the information about individual foods (e.g., generic foods or branded products), food coding systems (e.g., LanguaL) and/or standardized food classification and description system (e.g., Food EX2), nutrients or other food components reported, the number of food items contained in the tool (e.g., comprehensive food lists or specific foods rich in a specific nutrient or bioactive component), and features of the response section (e.g., whether eating occasions or time is recorded, if food groups are included). We recommend reporting not only the source of the food composition data, but also to report the number of nutrients it contains, the coverage, and how the tool has been customized to best meet the population-specific needs.

We recommend defining the context for the tool and report if (1) a targeted tool provides relative or absolute intake estimates and (2) whether you are estimating daily intakes, habitual total dietary intakes, or temporal intake changes. It is also important to report if a given tool queries about supplementary information on physical activity, health characteristics, or use of dietary supplements.

#### 4.1.3. Step 3: Report on the Appropriate Platform/Technology for the Tool

The goal of the third step is to report on the selection of the appropriate platform or technology of the tool. The choice for or against a specific technology type (e.g., tablet, computer, smartphone, wearable devices or multiple systems) depends strongly on the purpose and measures’ needs. Factors affecting this step are the available resources (i.e., financial, logistical and staff conditions). The level of technology-literacy of the targeted population needs to be taken in careful consideration. Other considerations include data sharing needs (i.e., how the participant/user data are exported and to whom), data storage structure and access, statistical analysis, programming language used for scripting the tool, how the individual will access the tool, and how their privacy will be maintained.

#### 4.1.4. Step 4: Report on the Customization Features of the Tool

The fourth step is to report on the customization of the features of the tool. These features, such as the type of data entry (e.g., text, voice, image capture, barcode scanning), list of foods and source of food composition data, type of portion size estimation (e.g., standardized portions, household measures or weights, pictures, automatic food volume estimations), need to be evaluated with respect to their adequacy to capture the purpose- and measures-specific needs of a given tool. One evaluation approach is to specifically assess the completeness and adequacy of the foods/recipes included in the tool in order to evaluate whether or how missing items could be added or recipes could be customized. Furthermore, the relevance of the dietary information in the output needs to be evaluated, as well as the need to provide feedback or to set goals for self-monitoring. Overall, details of the features that can be customized should be reported, and if there are any, an individual customization protocol should be developed and followed.

#### 4.1.5. Step 5: Report on the Design, Pretest, and Validation of the Tool

The fifth step is to report on the design and pre-test of the tool. User interface, tool format, wording and order of questions (as appropriate) as well as browsers and battery storage are likely to affect design features of the platform and technology tool. When studying culturally diverse populations, these aspects become even more important (e.g., does the wording have the same meaning in different languages). As with any dietary assessment method, technology tools should be pre-tested, ideally on a sample of subjects similar to those who will ultimately be studied. The purpose is to report on the ease of use or user friendliness and to identify questions that are poorly understood, ambiguous, or evoke implausible or other undesirable responses. We recommend reporting on the completion time and acceptability for implementing the tool. In addition, report how the tool has been validated and against what standard.

## 5. Conclusions

Dietary assessment methods that utilize technology provide rapid feedback to users and offer potential cost-savings for researchers. Dietary assessment methods that utilize new technology may be more appealing and engaging than paper-based methods, particularly for children and young adults. Online methods can be deployed to large groups with minimal resources compared with methods requiring in-field researchers. In addition, many of these tools provide rapid feedback to participants that may improve compliance with diet plans or research. Connectivity enables rapid and remote interaction with the participants and nutrition professionals or researchers. Combination methods may enhance the accuracy of dietary intake reporting (such as the use of digital images to improve memory and portion size estimates).

Many of the new technology tools assessed here showed close agreement to traditional methods of dietary intake, but gaps are wider when compared to more objective measures, like TEE from doubly-labelled water, though studies using this method are limited in number. We encourage developers and researchers to publish details about their dietary assessment tools, including those designed for consumer use, and call on the research community to evaluate the validity of the tools they create and use. While we were able to extract details about many features from the tools evaluated, it often required more than one publication to find the necessary information. We recommend that descriptions of tool development and features be clearly written in publications, covering all aspects of tool development, including data entry, food description, customization features, output characteristics, sources of food composition data, and results of usability and validity studies, following the guidance provided here.

## Figures and Tables

**Table 1:** Design characteristics of the technology-based tools used in dietary intake assessment.

Device Name	Country	Main Purpose of the Tool	Target Audience	Main Platform for Tool	Method of Data Collection and Entry	Food Composition Source	Approximate Number of Items	Time to Complete	References
Tools for Use in Research or Surveillance (*n* = 33)									
ASA24 Automated Self-Administered 24 h Recall	USA, Canada, Australia	Dietary intake	Adults and children from 10 years	Web-based	24-h recall based on automated multiple-pass method (AMPM)	USDA’s FNDDS ^4^, Canadian Food Composition and Australian food tables	10,000	Average of 24 min; most within 17–34 min	Baranowski et al. 2012 and 2014; Kirkpatrick et al. 2014; Thompson et al. 2015 [17,18,19,20]
CHAT Connecting Health and Technology; mobile food record	Australia	Food groups consumed	Adults and adolescents	Smartphone App	Food record based on images; dietitian identifies foods and food groups	Australia Guide to Healthy Eating, but not integrated into tool	2670	Not specified	Kerr et al. 2012 and 2016; Pollard et al. 2016 [21,22,23]
Compl-Eat	Netherlands	Dietary intake	Adults and adolescents from 16 years	Web-based	Interviewer-assisted or self-administered 24-h recall based on	Dutch Food Composition Database	2000	Close to 30 min	Meijboom et al. 2017 [24]
DAP Diet Assess and Plan	Serbia, Balkan region	Diet and physical activity	All ages	Web-based, PC	24-h recalls, food frequency questionnaires (FFQ), food propensity; dietitian enters data	Serbian and Balkan regional food composition databases	1450 Serbian and 1600 Balkan foods and recipes ^9^	15–30 min	Gurinovic et al. 2016 and 2018; Zekovic et al. 2017 [25,26,27]
DES Diet Evaluation System	Korea	Dietary intake	All ages	Web-based	Interviewer-assisted 24-h recall	Korean food composition tables	8100	Average of 14 min	Jung et al. 2014 [28]
eButton	USA	Dietary intake, activity	All ages	Wearable	Imaging system with automated portion estimates; dietitian enters data	USDA’s FNDDS, but not integrated into tool	8500	Not specified	Sun et al. 2010 and 2014; Jia et al. 2014 [29,30,31]
e-CA Electronic Carnet Alimentaire	Switzerland	Dietary intake	Adults	Web-based	Electronic food record; dietitian enters for coding	Prodi 6.3 software, but not integrated into the tool	900	Average of 19 min	Bucher Della Torre et al. 2017 [32]
eDIA Electronic Dietary Intake Assessment	Australia	Dietary intake	19–24 years old	Smartphone app	Food record	AUSNUT ^5^ 2007	4500	Not specified	Rangan et al. 2015 and 2016 [33,34]
EPIC-Soft ^1^	European Union (EU)	Dietary intake	All ages	PC with Web-based management platform	Interviewer-assisted 24-h recall or dietitian enters data from food records	EPIC ^6^ software from all EU countries	10,000	Not specified	de Boer et al. 2011; Huybrechts et al. 2011; Freisling et al. 2014; Park et al. 2017 [35,36,37,38]
Food4Me	EU—7 European counties	Dietary Intake	Adults	Web-based	FFQ	WISP ^7^ software; based on McCance and Widdowson	157 items grouped into 11 categories	Not specified	Fallaize et al., 2014; Forster et al., 2014; Celis-Morales et al. 2016 [39,40,41]
FoodBook24	Ireland	Dietary intake	Adults	Web-based	Food record, FFQ, food choice	Irish National Adult Nutrition Survey food composition database	751	Average of 15 min ^10^	Timon et al. 2017a and 2017b [42,43]
FoodNow	Australian	Diet and physical activity	Young adults	Smartphone; wearable armband for energy expenditure	Food record based on images, text or voice; dietitian enters data	2011–2013 Australian Food and Nutrient Database	5740	Not specified	Pendergast et al. 2017 [44]
GraFFS Graphical Food Frequency System	US	Dietary intake	Adults	Web-based	FFQ	NDSR and USDA’s FNDDS	156	Not specified	Kristal et al. 2014 [45]
INTAKE24 Self-Completed Recall and Analysis of Nutrition ^2^	UK	Dietary Intake	Adults and children from 11 years	Web-based	24-h recall based on AMPM	McCance and Widdowson	2800	Average of 13 min with flat interface	Foster et al. 2014; Bradley et al. 2016; Simpson et al. 2017 [46,47,48]
Microsoft SenseCam	Ireland, UK, Australia, others	Dietary intake, activity	Tested in athletes and different adult groups	Wearable	Imaging system to enhance 24-h recall interviews	WinDiets, but not integrated into tool	WinDiets has food databases from many countries	Not specified	O’Loughlin et al. 2013; Gemming et al. 2013 and 2015 [49,50,51]
myfood24	UK	Dietary Intake	Young Adults, Adults, Elderly	Web-based	24-h recall based on AMPM or food record	UK food composition database (branded and generic foods)	45,000	Average of 19 min (+/−7 min)	Carter et al. 2015; Albar et al. 2016 [52,53]
NINA-DISH New Interactive Nutrition Assistant	India: specifically New Delhi, Mumbai and Trivandrum	Dietary intake	Adults (35–69)	PC	Interviewer-assisted 24-h recalls, diet history, mealtime and food-preparer questionnaire	Indian FCT ^8^ augmented with data from UK, FNDDS, Singapore and Malaysia	1000	Not specified	Daniel et al. 2014 [54]
NANA Novel Assessment of Nutrition and Ageing	UK and USA	Dietary intake, activity, cognitive function	Elderly	Touch-screen computer with audio-recording	Food record based on images and voice; dietitian enters data	Windiets, but not integrated into tool	1200	Not specified	Astell et al. 2014; Timon et al. 2015 [55,56]
NuDAM Nutricam Dietary Assessment Method	Australia	Dietary intake	Adults	Smartphone/camera	Food record based on images and voice notes; dietitian enters data	FoodWorks 5.1, but not integrated into tool	13,000	Not specified	Rollo et al. 2011 and 2015 [57,58]
NutriNet Santé	France	Diet and physical activity	Adults	Web-based	24-h recall or food record based on AMPM	French food composition table	2600	Average of 31 ± 29 min; Median 25 min	Touvier et al. 2011 [59]
Oxford WebQ	UK	Diet and physical activity	Adults	Web-based, PC	24-h dietary checklist	McCance and Widdowson	200 items in 21 food groups	Average of 14 min; Median 12.5 min	Liu et al. 2011; Galante et al. 2016 [60,61]
R24W Self-Administered Web-based recall	French Canadian	Dietary intake	Adults and adolescents from 16 years	Web-based	24-h recalls based on AMPM	Canadian Nutrient file 2010 and Foods Commonly Consumed in Canada	4000	27.6% reported < 20 min, 31% 20–30 min, 24.1% 30–45 min, 7% 45–60 min	Jacques et al. 2016; Lafrenière et al. 2017 [62,63]
RFPM Remote Food Photography Method	USA	Dietary intake	All ages	Smartphone/camera/bar-code reader	Remote imaging system; semi-automated food identification	USDA’s FNDDS, but not integrated into tool	8500	Not specified	Martin et al. 2012 and 2014; Nicklas et al. 2017 [64,65,66]
SNAP Synchronized Nutrition and Activity Program	UK	Diet and physical activity	Children	Web-based	Food records collected during eight time-points daily	UK food consumption database	49 (40 foods, nine beverages)	<25 min	Moore et al. 2013 [67]
SNAPA Synchronized Nutrition and Activity Program for Adults	UK	Diet and physical activity	Adults	Web-based	Food records collected during 4 time periods each day	UK food consumption database	120 (102 foods and 18 beverages)	Not specified	Hillier, et al. 2012 [68]
TADA Technology Assisted Dietary Assessment;mobile food record	USA	Dietary intake	Adults and children from 3 years	Smartphone App	Food record based on before and after images of foods and beverages; system calculates energy and nutrients	USDA’s Food and Nutrient Database for Dietary Studies (FNDDS)	8500	Not specified	Daugherty et al. 2012; Ahmad et al. 2016; Boushey et al. 2015 and 2017 [69,70,71,72]
TECH Tool for Energy Balance in Children	Sweden	Diet and physical activity	2–5 years old	Smartphone App	Food record: Parents take images and provide short descriptions; dietitian enters data	Swedish Food Database, but this was not integrated into tool	Not reported	Not specified	Delisle et al. 2015; Henriksson et al. 2015; Delisle Nyström et al. 2016 [73,74,75]
VNP Virtual Nutri Plus	Brazil	Dietary intake	Patients undergoing gastric bypass surgery	PC	24-h recall or food record; dietitian enters data	Brazilian Food Chemical Composition Table	1711	Not specified	da Silva et al. 2014a and 2014b [76,77]
WebCAAFE Food Intake and Physical Activity of School-children	Brazil	Diet and physical activity	Children 6–12 years	Web-based	24-h recall	None; evaluates foods and beverages only	32 items in each of 6 eating events per day	Not specified	Davies et al., 2015; Kupek et al. 2016 [78,79]
WebDASC Web-Based Dietary Assessment Software for Children	Denmark	Dietary Intake	Children	Web-based	24-h recall	Danish National Survey of Diet and Physical Activity (DANSDA)	1300	Average of 15 min (after first day)	Biltoft-Jensen et al. 2012 and 2013; Andersen et al. 2015 [80,81,82]
Web-FFQ	Quebec, Canada	Dietary intake	Adults	Web-based	FFQ	Nutrition Data System for Research and the Canadian Nutrient File	136	45 min	Labonte et al. 2012 [83]
WebFR Web-based Food Record	Norway	Dietary Intake	Children	Web-based	24-h recall	Norwegian National Survey database (NORKOST)	550	Not specified	Medin et al. 2015, 2016, and 2017 [84,85,86]
Zambia Tablet-based 24h recall Tool	Zambia	Dietary intake	Children	Tablet	Interviewer-assisted 24-h recall	HarvestPlus and Zambia food comp tables	Not specified	Not specified	Caswell et al. 2015 [87]
Tools for Consumer Use (*n* = 10)									
Diabetics Diary, paired with Pebble smartwatch	Norway	Diabetes management Diet and physical activity	Adults	Android Smartphone plus Smart watch	Carbohydrate food log	None	Not reported	Not specified	Arsand et al. 2015 [88]
DietCam	USA	Dietary intake	All ages for obesity prevention	Smartphone App	Food record from images; system calculates energy	USDA National Nutrient Database for Standard Reference	8500	Not specified	Kong and Tan, 2011 and 2012; Kong thesis, 2012; Kong et al. 2015 [16,89,90,91]
DIMA Dietary Intake Monitoring Application	USA	Medical management and diet	Hemodialysis patients	PDA	Food record with touch, voice, bar-code scanner	Database was created from existing nutrient database and UPC codes	Not specified	Not specified	Connelly et al. 2012; Welch et al. 2013 [92,93]
EVIDENT II app	Spain	Adherence to a Med Diet and log for step counter	Adults	Smartphone App	FFQ and Med Diet checklist	Spanish FFQ	137s	Not specified	Recio-Rodriguez et al. 2014 and 2016 [94,95]
FoodLog for dietary data collection as part of DialBetics program	Japan, Korea	Diabetes management and diet, physical activity	Adults	Smartphone App	Food record from images, text; system calculates energy, macro-nutrients	National food database: Dietary Reference Intakes, Japan (2010)	2191	Average of 35 min	Aizawa K. et al. 2014; Waki et al. 2012 and 2015 [96,97,98]
GoCARB	EU	Diabetes management and diet	Adults	Smartphone App	Food record based on meal images for carbohydrate intake estimates	USDA Nutrient Database for Standard Reference	5000	~1 min per image	Rhyner et al. 2016; Bally et al. 2017 [99,100]
IDQC Internet Based Diet and Lifestyle Questionnaire	China	Diet and physical activity	Adults and adolescents	Web-based	FFQ	Food Nutrition Calculator (Beijing)	135	30–40 min	Du et al. 2015 [101]
My Meal Mate (MMM)	UK	Diet, activity and body weight	Adults—Weight loss or maintenance	Smartphone App	Food record	UK Composition of Foods	40,000	Average of 22 min	Carter et al. 2013a, 2013b, 2013c [102,103,104]
Snap-n-Eat mobile application	USA	Dietary intake	Adults	Smartphone App	Food record from images; system calculates energy and nutrients	not reported	not reported	Not specified	Zhang et al. 2015 [15]
SuperTracker ^3^	USA	Diet and physical activity	All ages	Web-based	Food records, diet recall	USDA’s FNDDS	8500	Not specified	Post et al. 2012; Tsompanakis, 2015 [105,106]

^1^ Now called Globo-Diet. ^2^ Formerly called SCRAN24, which was a PC-based platform. ^3^ Formerly called MyPyramid Tracker; discontinued as of July 2018. (Long et al., 2012 was for MyPyramid Tracker, the predecessor of SuperTracker). ^4^ FNDDS is the US Department of Agriculture’s Food and Nutrient Database for Dietary Studies; FNDDS provides the nutrient values for foods and beverages reported in the dietary intake component of the National Health and Nutrition Examination Survey (NHANES). ^5^ Australian Food, Supplement and Nutrient Database (AUSNUT). ^6^ European Prospective Investigation into Cancer and Nutrition (EPIC). ^7^ WISP (Tinuviel Software) is nutritional analysis software for the UK and Ireland (http://www.tinuvielsoftware.co.uk/wisp4.htm). ^8^ Food Composition Table (FCT). ^9^ Based on personal communication with M. Gurinović, University of Belgrade, Serbia. ^10^ Based on personal communication with S. Pigat, CremeGlobal, Dublin, Ireland.

## Supplementary Materials

The following are available online at http://www.mdpi.com/2072-6643/11/1/55/s1, Table S1. Search strategies used to identify technology-based tools for dietary intake assessment; Table S2. Details of data extraction and evaluation criteria used to evaluate new technology tools for dietary intake assessment; Table S3. Validation methods for total energy and macronutrients for the technology-based tools used in dietary intake assessment.
