# Supplementary material for: Evaluation of New Technology-Based Tools for Dietary Intake Assessment—An ILSI Europe Dietary Intake and Exposure Task Force Evaluation
Source: Nutrients. 2018 Dec 28;11(1):55. doi: 10.3390/nu11010055 (PMC6356426; doi:10.3390/nu11010055)
Supplement: Supplementary file 1 [file nutrients-11-00055-s001.pdf]

Supplementary Materials

Table S1. Search strategies used to identify technology-based tools for dietary intake assessment

Search 1 [1,2]	Search Terms (number of results)[1]
PubMed	Application + dietary assessment + electronic (26)<br>Application + dietary assessment + software (18)<br>Personal digital assistant + food record (19)<br>Dietary assessment + software + tools (148) current search<br>Application + dietary assessment + dietary intake (83)<br>Application + nutrition + technology (141)<br>Application + dietary intake (290)<br>Application + nutrition assessment + software (19)<br>Mobile application + dietary assessment (20)<br>Application + personal digital assistant + nutrition (8)<br>Application + dietary intake + web (13)
PLOS	Application + dietary assessment + electronic + software + mobile (470)<br>Application + nutrition assessment tool + software + food record + health + technology + management + mobile (140)<br>Application + nutritional assessment + dietary assessment + software + mobile + personal (339)<br>Application + dietary assessment + management + chronic disease + software + mobile (512)
BioMED	'Application + nutritional assessment + electronic + software + mobile' (103)<br>'Application + dietary assessment + dietary intake + software + mobile' (88)
Science Direct	nutritional assessment + software + apps (208)<br>Application + dietary assessment + dietary intake + software + mobile (501)
Search 2 [1,3]	
OVID	1. exp nutrition assessment/ (8916)<br>2. Nutrition surveys/ or diet surveys (11488)<br>3. Diet records/ (4291)<br>4. (diet* adj2 (recall* or questionnaire* or history* or instrument*)).tw. (5582)<br>5. (nutrition* adj2 (survey* or assess* or instrument*)).tw. (14782)<br>6. (food adj2 (questionnaire* or record* or recall* or diar* or checklist* or screener*)).tw. (11389)<br>7. 1 or 2 or 3 or 4 or 5 or 6 (40952)<br>8. exp internet/ (64354)<br>9. technolog*.tw. (251969)<br>10. web.tw. (59263)<br>11. online.tw. (51470)<br>12. (mobile adj2 app*).tw. (1540)<br>13. tool*.tw. (411302)<br>14. 8 or 9 or 10 or 11 or 12 or 13 (743498)<br>15. 7 and 14 (2938)<br>16. limit 15 to (english language and yr="2011 –Current") (1615)
Search 3 [1,3]	
OVID	Nutrition assessment plus diet* intake OR food intake plus valid*

	Excluding references included in previous OVID search (134)

[1] Inclusion criteria: English language publications

[2] Search conducted 1 August 2016 and included articles from 16 September 2011 to 31 July 2016.

[3] Search conducted 12 September 2017 and included articles from 1 January 2011 to 12 September 2017.

Table S2. Details of data extraction and evaluation criteria used to evaluate new technology tools for dietary intake assessment [1]

Category	Attribute	Data capture
GENERAL ATTRIBUTES	Device name	Tool name
	Location	Tool designed for use in which country or countries
	Main purpose of the tool	Dietary intake; Dietary intake and physical activity; Medical management
	Target audience	Ages and attributes
	Context	Research/surveillance; consumer
	Main platform of the tool	Web-based; Smartphone; wearable; Stand-alone personal computer (PC); multiple; PDA
	Type of data collected	Food record/recall; FFQ; Other
DATA ENTRY	Text	Subject enters data as text; interviewer or dietitian enters text data from participant; no text entry
	Photo	Self-recording via image capture (yes, no, not specified)
	Barcode	Data entry by bar-code scanner (yes, no, not specified)
	Health characteristics	Subject can record health characteristics, e.g. height, weight, age, medical measurements, etc. (yes, no, not specified)
	Physical activity	Subject can monitor their physical activity (yes with automated data transfer from another device, yes with manual data entry, no, not specified)
	Set personal goals	Subject can set personal goals (yes, no, not specified)
IDENTIFICATION and QUANTIFICATION of FOODS	Automated identification	Tool could automatically identify foods from images
	Manual identification of foods	Subject or dietitian manually selected food items
	Comprehensive national food composition database	Name of database and number of food items
	Intakes quantified by weights/ household measures	Yes, no, not specified
	Intakes estimated from digital images	Automatically, manually, or standards amounts assigned from images of portions of different size
CUSTOMIZATION	Ability to add missing foods	Yes, no, not specified

	Ability to add custom recipes	Yes, no, not specified
	Ability to record dietary supplements	Yes, no, not specified
	Learning system adapts food list	Yes, no, not specified
OUTPUT	Energy	Yes, no, not specified
	Macronutrients	Yes, no, not specified
	Micronutrients	Yes with extensive list, yes with limited list, no, not specified
	Food groups	Yes, no, not specified
	Time of intake	Yes, no, not specified
	Meal name	Yes, no, not specified
	Automated reports generated	Feedback to participants available from automated reports; feedback from manual reports; no feedback
USABILITY and VALIDITY	Usability feedback collected	Yes, no, not specified
	Time to complete reported	Yes plus time to record, no, not specified
	Validation studies done	Yes and method used, no, not specified

[1] Data extraction was also done to assess cost and whether data security was mentioned, but few tools reported on these attributes, so they were not included in the final assessment.

Table S3. Validation methods for total energy and macronutrients for the technology-based tools used in dietary intake assessment

Tool name	Type	Validation Method	Subjects	Energy	Significance Level	Protein, Fat, Carbohydrate	References
**Comparison of Estimated Energy Intakes from Technology Tools with Total Energy Expenditure (TEE) from DLW or Accelerometers**							
FoodNow	Research	TEE estimated from accelerometer	56 young adult men and women	10,030 ± 2210 kJ/d (TEE) v. 9204 ± 1958 kJ/d (FoodNow)	not reported		Pendergrast et al. 2017 [1]
Microsoft SenseCam	Research	TEE by DLW compared to 24-h recall plus SenseCam	20 men age 34.8 ± 12.6 y	14,485 ± 2632 kJ/d (TEE) v. 13,196 ± 2529 kJ/d (SenseCam + recall)	*P* = 0.02		Gemming et al. 2015 [2]
			20 women age 27.1 ± 7.5 y	10,841 ± 1639 kJ/d (TEE) v. 10,091 ± 1672 kJ/d (SenseCam + recall)	*P* = 0.004		Gemming 2015 et al. [2]
NuDAM	Research	TEE by DLW	10 men and women with Type 2 diabetes, 48-69 y old	11,800 ± 2300 kJ/d (TEE) v. 8800 ± 2000 kJ/d (Nutricam)	*P*< 0.01		Rollo et al. 2015 [3]
RFPM	Research	TEE by DLW	9 men and 31 women	10,314 ± 2330 kJ/d (TEE) v. 6569 ± 2261 (RFPM standard prompts, n=22)); 9109 ± 2054 (TEE) v. 7979 ± 2243 (RFPM custom prompts, n=13)	*P*< .001<br>*P* = 0.22		Martin et al. 2012 [4]
		TEE by DLW	6 men, 44 women	9874 ± 2619 (TEE free-living) v. 9238 ± 2782 (RFPM)	*P* = 0.16		Martin et al. 2012 [4]

TADA	Research	TEE by DLW, estimated by image review and from returned portions	15 men age 32 ± 9 y	Men: 14,846 kJ/d (TEE) v. 11,279 (image review) and 11,036 (returned portions)	*P*< 0.0001 TEE v. image review		Boushey et al. 2017 [5]
			30 women age 33 ± 13 y	Women: 10,995 kJ/d (TEE) v. 9136 (image review) and 9131 (returned portions)	*P*< 0.0001 v. image review		Boushey et al. 2017 [5]
TECH	Research	TEE by DLW	30 Swedish children age 3 y	5070 ± 600 kJ/24H (TEE) v. 5400 ± 1500 kJ/24h (TECH)	*P* = 0.23		Henriksson et al. 2015 [6]
		TEE by DLW and 24-h recall	39 Swedish children 5.5 ± 5 y	5820 ± 820 kJ/d (TEE) v. 6040 ± 680 kJ/d (TECH)	*P* = 0.06		Delisle Nystrom et al. 2016 [7]
WebFR	Research	TEE estimated from accelerometer data combined with child weight and sex	253 children ages 8-14 y	8690 ± 1310 kJ/d (TEE) v. 6850 ± 2130 kJ/d WebFR	not reported		Medin et al. 2017 [8]
**Comparisons of Estimated Energy Intakes from Technology Tools with Diet Recalls, Diet Records, or Controlled Feeding Studies**							
ASA 24	Research	Two 24-h recalls	512 men ages 20-70 y	10,153 kJ/d (24-h recall) v. 9939 kJ/d (ASA 24)	not reported	Intakes with 24-h recall v. ASA 24<br>Protein +3.3g<br>Fat -3.2g<br>CHO +6.0g	Thompson et al. 2015 [9]
			569 women ages 20-70 y	7854 kJ/d (24-h recall) v. 7980 kJ/d (ASA 24)	not reported	Intakes with 24-h recall v. ASA 24<br>Protein +4.0g<br>Fat +7.3g<br>CHO +3.3g	Thompson et al. 2015 [9]

Compl-Eat	Research	Three 24-h recalls	514 men and women ages 20-70 y	8728 ± 1947 kJ/d (24-h recall) v. 8014 ± 2122 kJ/d (Compl-Eat)	*P*< 0.0001	Intakes with 24-h recalls v. Compl-Eat<br>Protein +7.4g<br>Fat +9.1g<br>CHO +13.0g	Meijboom et al. 2017 [10]
e-CA	Research	Two 24-h recalls	18 men and women ages 20-60 y	9998 ± 2780 kJ/d (24-h recalls) v. 9575 ± 3316 kJ/d (e-CA)	Not statistically different	Intakes with 24-h recalls v. e-CA<br>Protein -2g<br>Fat +9g<br>CHO 0g	Bucher Della Torre et al. 2017 [11]
eDIA	Research	Three 24-h recalls	80 university students ages 19-24 y	8182 ± 2575 kJ/d (24-h recalls) v. 8148 ± 2495 kJ/d (eDIA)	not reported	Intakes with 24-h recalls v. eDIA<br>Protein +2.6 g<br>Fat +1.4g<br>CHO -22.3g	Rangan et al. 2015 [12]
Food4Me	Research	Four-day weighed food record	49 males and females, age 26.9 ± 8.4 y	8110 ± 2119 kJ/d (WFR) v. 8855 ± 3387 kJ/d (Food4Me)	*P* = 0.008	Intakes with WFR v. Food4Me:<br>Protein -0.4g<br>Fat -11.0g<br>CHO -10.0g	Fallaise et al. 2014 [13]
Foodbook 24	Research	24-h recall	79 men and women age 33.2 ± 12.5 y	8453 ± 2675 kJ/d (24-h recall) v. 7607 ± 2805 kJ/d (Foodbook 24)	not reported	Intakes with 24-h recall v. Foodbook 24<br>Protein +7g<br>Fat +11g<br>CHO +17g	Timon et al. 2017 [14]

GraFFS	Research	Six 24-h recalls	74 men and women ages 18-69 y	8235 (CI 5334-12,606) kJ/d (24-h recalls) v. 7201 (CI 3542-14,645) kJ/d (GraFFS)	*P*< 0.02	Intakes with 24-h recalls v. GraFFS<br>Protein +13.1g<br>Fat +11.5g<br>CHO +20.9g	Kristal et al. 2014 [15]
IDQC	Consumer	3-d food diary	644 male and female college students	8545 kJ/d (Diary) v. 9412 kJ/d (IDQC)	*P*< 0.05	Intakes with 3-d diary v. IDQC<br>Protein -9.0g<br>CHO -21.2g	Du et al. 2015 [16]
Intake24	Research	Four 24-h recalls	52 boys and girls ages 11-16 y	6824 kJ/d (24-h recalls) v. 6682 kJ/d (Intake24)	not reported	Intakes with 24-h recalls vs. Intake24<br>Protein 0.0g<br>Fat +3.5g<br>CHO +1.8g	Bradley et al. 2016 [17]
		Four 24-h recalls	116 boys and girls ages 17-24 y	7516 kJ/d (24-h recalls) v. 7408 kJ/d (Intake24)	not reported	Intakes with 24-h recalls vs. Intake24<br>Protein -1.3g<br>Fat -0.4g<br>CHO +1.2g	Bradley et al. 2016 [17]
My Food 24	Research	Two 24-h recalls	75 adolescents, ages 11-18 y	Day 1: 8745 ± 3814 kJ/d (24-h recall) v. 8514 ± 4020 kJ/d (MyFood24)<br>Day 2: 8035 ± 2561 kJ/d (24-h recall) v. 7820 ± 2746 kJ/d (MyFood24)	*P* = 0.40	Intakes with 24-h recalls v. MyFood24:<br>Protein +1.2g<br>Fat +2.9g<br>CHO +11.1g	Albar et al. 2016 [18]

MyMealMate	Consumer	Two 24-h recalls	50 men and women, ages 35 ± 9 y	8401 ± 2050 (24-h recalls) v. 8196 ± 2146 kJ/d (MMM)	*P* = 0.23	24-h recalls v. MyMealMate:<br>Protein +2.5g<br>Fat +3.0g<br>CHO +5.0g	Carter et al. 2013 [19]
			50 men and women, ages 35 ± 9 y	8242 ± 1686 (24-h recalls) v. 8020 ± 1695 kJ/d (MMM)	*P* = 0.30		Carter et al. 2013 [19]
NANA	Research	Four-day food diary	40 adults ages 65-89 y	7348 ± 1503 kJ/d (Diary) v. 7098 ± 1382 kJ/d (NANA)	*P* = 0.048	Intakes with Food Diary v. NANA:<br>Protein +3g<br>Fat -1g<br>CHO +7g	Astell et al. 2014 [20]
			94 adults ages 65-89 y	7709 ± 177 kJ/d (Diary) v. 7461 ± 158 (NANA)	*P* = 0.004	Intakes with Food Diary v. NANA:<br>Protein +3.7g<br>Fat -0.9g<br>CHO +5.2g	Timon et al. 2015 [21]
NuDAM	Research	3-d food diary	10 men and women with Type 2 diabetes, 59-70 y old	6946 ± 1837 (food diary) v. 6297 ± 1964 kJ/d (NuDAM)	*P* ≤ 0.05		Rollo et al. 2011 [22]
		Weighed food records	10 men and women with Type 2 diabetes, 48-69 y old	8800 ± 1800 kJ/d (WFR) v. 8800 ± 2000 kJ/d (NuDAM) – part of same study as DLW above	not significant	WFR v. Nutricam<br>Protein -4.7g<br>Fat -1.2g<br>CHO +1.1g	Rollo et al. 2015 [3]

Nutrinet Santé	Research	One 24-h recall	60 men ages 48-75 y	8993 ± 2286 kJ/d (24-h recall) v. 8848 ± 2583 kJ/d (Nutrinet Santé)	not reported	Intakes with 24-h recall v. Nutrinet Santé<br>Protein -2.2g<br>Fat +5.0g<br>CHO -3.3g	Touvier et al. 2011 [23]
			87 women ages 48-75 y	7182 ± 2080 kJ/d (24-h recall) v. 7204 ± 2467 kJ/d (Nutrinet Santé)	not reported	Intakes with 24-h recall v. Nutrinet Santé<br>Protein -1.0<br>Fat +1.7<br>CHO -4.2g	Touvier et al. 2011 [23]
Oxford WebQ	Research	One 24-h recall	116 men and women, ages 19-82 y	8702 ± 2600 kJ/d (24-h recall) v. 8713 ± 2463 kJ/d (Oxford WebQ)	not reported	Intakes with 24-h recalls v. Oxford WebQ:<br>Protein +1.0g<br>Fat -3.5g<br>CHO +5.4g	Liu et al. 2011 [24]
R24W	Research	Two days controlled feeding	62 men and women ages 18-75 y	11,624 ± 2528 kJ/d (controlled feeding) v. 11,566 ± 3270 kJ/d (R24W)	Not significant at $P \leq 0.05$ level	Intakes with controlled feeding v. R24W<br>Protein -0.9g<br>Fat -8.2g<br>CHO +26.0g	Lafenière et al. 2017 [25]
RFPM	Research	Weighed buffet meal	49 men and women	2456 ± 874 kJ/d (weighed buffet) v. 2439 ± 795 kJ/d (RFPM)	$P = 0.67$	Intakes from weighed buffet vs. RFPM<br>Protein -1.5g<br>Fat 0.8g<br>CHO -4.3g	Martin et al., 2012 [4]

TECH	Research	24-h recall	39 Swedish children 5.5 ± 5 y	5990 ± 680 kJ/d (24-h recall) v. 6040 ± 680 kJ/d (TECH)	$P = 0.60$		Delisle Nystrom et al. 2016 [7]
Web-FFQ	Research	3-d food diary	69 men and women age 37.1 ± 14.2 y	9377 ± 2232 kJ/d (Diary) v. 9477 ± 2941 kJ/d (Web-FFQ)	$P = 0.76$	Intakes with 3-d diary v. Web-FFQ<br>Protein +1.3g<br>Fat -2.4g<br>CHO -5.3g	Labonte et al. 2012 [26]

References for Supplementary Materials (all also appear in the references for the main text, but with different numbering)

1. Pendergast, F.J.; Ridgers, N.D.; Worsley, A.; McNaughton, S.A. Evaluation of a smartphone food diary application using objectively measured energy expenditure. *Int J Behav Nutr Phys Act* **2017**, *14*, 30, doi:10.1186/s12966-017-0488-9.
2. Gemming, L.; Rush, E.; Maddison, R.; Doherty, A.; Gant, N.; Utter, J.; Ni Mhurchu, C. Wearable cameras can reduce dietary under-reporting: doubly labelled water validation of a camera-assisted 24 h recall. *Br J Nutr* **2015**, *113*, 284-291, doi:10.1017/S0007114514003602.
3. Rollo, M.E.; Ash, S.; Lyons-Wall, P.; Russell, A.W. Evaluation of a mobile phone image-based dietary assessment method in adults with Type 2 Diabetes. *Nutrients* **2015**, *7*, 4897-4910, doi:10.3390/nu7064897.
4. Martin, C.K.; Correa, J.B.; Han, H.; Allen, H.R.; Rood, J.C.; Champagne, C.M.; Gunturk, B.K.; Bray, G.A. Validity of the Remote Food Photography Method (RFPM) for estimating energy and nutrient intake in near real-time. *Obesity (Silver Spring)* **2012**, *20*, 891-899, doi:10.1038/oby.2011.344.
5. Boushey, C.J.; Spoden, M.; Delp, E.J.; Zhu, F.; Bosch, M.; Ahmad, Z.; Shvetsov, Y.B.; DeLany, J.P.; Kerr, D.A. Reported energy intake accuracy compared to doubly labeled water and usability of the Mobile Food Record among community dwelling adults. *Nutrients* **2017**, *9*, doi:10.3390/nu9030312.
6. Henriksson, H.; Bonn, S.E.; Bergstrom, A.; Balter, K.; Balter, O.; Delisle, C.; Forsum, E.; Lof, M. A new mobile phone-based tool for assessing energy and certain food intakes in young children: a validation study. *JMIR Mhealth Uhealth* **2015**, *3*, e38, doi:10.2196/mhealth.3670.
7. Delisle Nystrom, C.; Forsum, E.; Henriksson, H.; Trolle-Lagerros, Y.; Larsson, C.; Maddison, R.; Timpka, T.; Lof, M. A mobile phone based method to assess energy and food intake in young children: a validation study against the doubly labelled water method and 24 h dietary recalls. *Nutrients* **2016**, *8*, doi:10.3390/nu8010050.
8. Medin, A.C.; Hansen, B.H.; Astrup, H.; Ekelund, U.; Frost Andersen, L. Validation of energy intake from a web-based food recall for children and adolescents. *PLoS One* **2017**, *12*, e0178921, doi:10.1371/journal.pone.0178921.
9. Thompson, F.E.; Dixit-Joshi, S.; Potischman, N.; Dodd, K.W.; Kirkpatrick, S.I.; Kushi, L.H.; Alexander, G.L.; Coleman, L.A.; Zimmerman, T.P.; Sundaram, M.E., et al. Comparison of interviewer-administered and automated self-administered 24-hour dietary recalls in 3 diverse integrated health systems. *Am J Epidemiol* **2015**, *181*, 970-978, doi:10.1093/aje/kwu467.

10. Meijboom, S.; van Houts-Streppel, M.T.; Perenboom, C.; Siebelink, E.; van de Wiel, A.M.; Geelen, A.; Feskens, E.J.M.; de Vries, J.H.M. Evaluation of dietary intake assessed by the Dutch self-administered web-based dietary 24-h recall tool (Compl-eat) against interviewer-administered telephone-based 24-h recalls. *J Nutr Sci* **2017**, *6*, e49, doi:10.1017/jns.2017.45.
11. Bucher Della Torre, S.; Carrard, I.; Farina, E.; Danuser, B.; Kruseman, M. Development and evaluation of e-CA, an electronic mobile-based food record. *Nutrients* **2017**, *9*, doi:10.3390/nu9010076.
12. Rangan, A.M.; O'Connor, S.; Giannelli, V.; Yap, M.L.; Tang, L.M.; Roy, R.; Louie, J.C.; Hebden, L.; Kay, J.; Allman-Farinelli, M. Electronic Dietary Intake Assessment (e-DIA): comparison of a mobile phone digital entry app for dietary data collection with 24-hour dietary recalls. *JMIR Mhealth Uhealth* **2015**, *3*, e98, doi:10.2196/mhealth.4613.
13. Fallaize, R.; Forster, H.; Macready, A.L.; Walsh, M.C.; Mathers, J.C.; Brennan, L.; Gibney, E.R.; Gibney, M.J.; Lovegrove, J.A. Online dietary intake estimation: reproducibility and validity of the Food4Me food frequency questionnaire against a 4-day weighed food record. *J Med Internet Res* **2014**, *16*, e190, doi:10.2196/jmir.3355.
14. Timon, C.M.; Evans, K.; Kehoe, L.; Blain, R.J.; Flynn, A.; Gibney, E.R.; Walton, J. Comparison of a Web-based 24-h dietary recall tool (Foodbook24) to an interviewer-led 24-h dietary recall. *Nutrients* **2017**, *9*, doi:10.3390/nu9050425.
15. Kristal, A.R.; Kolar, A.S.; Fisher, J.L.; Plascak, J.J.; Stumbo, P.J.; Weiss, R.; Paskett, E.D. Evaluation of web-based, self-administered, graphical food frequency questionnaire. *J Acad Nutr Diet* **2014**, *114*, 613-621, doi:10.1016/j.jand.2013.11.017.
16. Du, S.S.; Jiang, Y.S.; Chen, Y.; Li, Z.; Zhang, Y.F.; Sun, C.H.; Feng, R.N. Development and applicability of an internet-based diet and lifestyle questionnaire for college students in China: a cross-sectional study. *Medicine (Baltimore)* **2015**, *94*, e2130, doi:10.1097/MD.0000000000002130.
17. Bradley, J.; Simpson, E.; Poliakov, I.; Matthews, J.N.; Olivier, P.; Adamson, A.J.; Foster, E. Comparison of INTAKE24 (an online 24-h dietary recall tool) with interviewer-led 24-h recall in 11-24 year-old. *Nutrients* **2016**, *8*, doi:10.3390/nu8060358.
18. Albar, S.A.; Alwan, N.A.; Evans, C.E.; Greenwood, D.C.; Cade, J.E. Agreement between an online dietary assessment tool (myfood24) and an interviewer-administered 24-h dietary recall in British adolescents aged 11-18 years. *Br J Nutr* **2016**, *115*, 1678-1686, doi:10.1017/S0007114516000593.
19. Carter, M.C.; Burley, V.J.; Nykjaer, C.; Cade, J.E. 'My Meal Mate' (MMM): validation of the diet measures captured on a smartphone application to facilitate weight loss. *Br J Nutr* **2013**, *109*, 539-546, doi:10.1017/S0007114512001353.
20. Astell, A.J.; Hwang, F.; Brown, L.J.; Timon, C.; Maclean, L.M.; Smith, T.; Adlam, T.; Khadra, H.; Williams, E.A. Validation of the NANA (Novel Assessment of Nutrition and Ageing) touch screen system for use at home by older adults. *Exp Gerontol* **2014**, *60*, 100-107, doi:10.1016/j.exger.2014.10.008.
21. Timon, C.M.; Astell, A.J.; Hwang, F.; Adlam, T.D.; Smith, T.; Maclean, L.; Spurr, D.; Forster, S.E.; Williams, E.A. The validation of a computer-based food record for older adults: the Novel Assessment of Nutrition and Ageing (NANA) method. *Br J Nutr* **2015**, *113*, 654-664, doi:10.1017/S0007114514003808.
22. Rollo, M.E.; Ash, S.; Lyons-Wall, P.; Russell, A. Trial of a mobile phone method for recording dietary intake in adults with type 2 diabetes: evaluation and implications for future applications. *J Telemed Telecare* **2011**, *17*, 318-323, doi:10.1258/jtt.2011.100906.

23. Touvier, M.; Kesse-Guyot, E.; Mejean, C.; Pollet, C.; Malon, A.; Castetbon, K.; Hercberg, S. Comparison between an interactive web-based self-administered 24 h dietary record and an interview by a dietitian for large-scale epidemiological studies. *Br J Nutr* **2011**, *105*, 1055-1064, doi:10.1017/S0007114510004617.
24. Liu, B.; Young, H.; Crowe, F.L.; Benson, V.S.; Spencer, E.A.; Key, T.J.; Appleby, P.N.; Beral, V. Development and evaluation of the Oxford WebQ, a low-cost, web-based method for assessment of previous 24 h dietary intakes in large-scale prospective studies. *Public Health Nutr* **2011**, *14*, 1998-2005, doi:10.1017/S1368980011000942.
25. Lafrenière, J.; Benoît Lamarche, B.; Catherine Laramée, C.; Julie Robitaille, J.; Lemieux, S. Validation of a newly automated webbased 24-hour dietary recall using fully controlled feeding studies. *BMC Nutr.* **2017**, *3*, doi:DOI 10.1186/s40795-017-0153-3.
26. Labonte, M.E.; Cyr, A.; Baril-Gravel, L.; Royer, M.M.; Lamarche, B. Validity and reproducibility of a web-based, self-administered food frequency questionnaire. *Eur J Clin Nutr* **2012**, *66*, 166-173, doi:10.1038/ejcn.2011.163.
